# Fungi and Insect Interactions: Pathogenicity, Immune Defenses and Biocontrol

**DOI:** 10.3390/jof11040289

**Published:** 2025-04-07

**Authors:** Jiaqin Xie, Nicolás Pedrini

**Affiliations:** 1 School of Life Sciences, Genetic Engineering Research Center, Chongqing University, Chongqing 400044, China; 2 Instituto de Investigaciones Bioquímicas de La Plata (INIBIOLP), CCT La Plata Consejo Nacional de Investigaciones Científicas y Técnicas (CONICET), Universidad Nacional de La Plata (UNLP), Calles 60 y 120, La Plata 1900, Argentina

## 1. Introduction

Insects are the largest animal group in nature, with significant effects on ecology, human health, and indigenous flora and fauna. Naturally abundant insect-pathogenic (i.e., entomopathogenic) fungi play a crucial role in regulating insect populations and have complex interactions with them. Entomopathogenic fungi infect insects by attaching to, germinating on, and penetrating the host’s exoskeleton, ultimately proliferating within the insect’s body and tissues. In response, host insects have evolved physical barriers, immune responses, microbiota, and behavioral defenses against fungal infections. Furthermore, insect-pathogenic fungi are valuable in biological pest management, as they often lead to the death of their hosts. They can also act as facultative saprophytes in the soil and enhance plant resistance to herbivores as endophytes. While the general mechanisms of the interaction between fungi and insects are well understood, many aspects at the molecular, physiological, and behavioral levels remain unclear. Therefore, studying the interaction between fungi and insects in this specific context could provide valuable and extensive insights.

These topics were addressed in the Special Issue “Fungi and Insect Interactions: Pathogenicity, Immune Defenses and Biocontrol”, comprising 13 original studies focused on the biological and molecular aspects of the interaction between entomopathogenic fungi and their wide array of hosts, including arthropods and plants, which we briefly summarize in the following paragraphs and encourage readers to explore in full.

## 2. An Overview of Published Articles

The study by Chacón-Fuentes et al. (Contribution 1) reported a countermeasure strategy employed by the Chilean pest *Chilesia rudis* against peramine, often produced by fungi. This pest larva has evolved the ability to adapt to endophyte-infected ryegrass and develop counter-adaptation mechanisms to mitigate the side effects of such alkaloids. In their research, Wang et al. (Contribution 2) aimed to enhance the efficiency of the entomopathogenic fungus *Metarhizium rileyi* in targeting insect pests, addressing its slow growth rate, low resistance to abiotic stress, and slow killing speed by incorporating various lipids. Their findings revealed that exogenous oleic acid and linoleic acid could boost stress tolerance and virulence of *M. rileyi* by safeguarding conidial germination and facilitating cuticle infection. Su et al. (Contribution 3) identified the catalase gene *MrCat1* as a key player influencing various functions of the entomopathogenic fungus *M. rileyi*, including oxidative stress tolerance, microsclerotia formation, and virulence. Furthermore, Song et al. (Contribution 4) and Hong et al. (Contribution 5) reported on the negative regulation of UV- and thermo-tolerances by the forkhead box gene *MaSep1* and the role of a new transcription factor *MaAzaR* in influencing virulence to *Locusta migratoria manilensis* through cuticle penetration in *Metarhizium acridum*, respectively. Chen et al. (Contribution 6) highlighted the significant potential of two isolated *Beauveria bassiana* strains in controlling rice pests *Nilaparvata lugens* and *Sogatella furcifera*. A subsequent study suggested that combining these strains with chlorfenapyr or dinotefuran could enhance control efficacy against these pests. The study by Mao et al. (Contribution 7) examined the effects of three insecticides and three fungicides on the mycelial growth and spore germination of *Cordyceps javanica*. They also investigated the impact of combining these chemical pesticides with the fungus against *Myzus persicae* to enhance the efficiency of mycoinsecticides in biocontrol strategies. Preisegger et al. (Contribution 8) utilized a conidial hydrogel formulation of GFP-tagged *B. bassiana* to study per os infection and track the conidial movement through the alimentary canal of the red flour beetle, *Tribolium castaneum*. They found that ingested conidia are unable to germinate within the midgut but have a measurable impact on the insects’ fitness, increasing their susceptibility to fungal infection and eventual mortality. Wang et al. (Contribution 9) utilized homologous recombination to create deletion mutants and complementation strains of the nitrate transporter MaNrtB in *M. acridum*. They found that the disruption strain was more vulnerable to various stressors and exhibited impaired fungal virulence, characterized by reduced appressorium formation on the cuticle and attenuated growth in the hemolymph of *Locusta migratoria manilensis.* The study by Boaventura et al. (Contribution 10) provides a comprehensive understanding of the effects of both constant and fluctuating temperatures on the growth and virulence of the entomopathogenic fungus *C. javanica* against the whitefly *Bemisia tabaci*. Their results, based on fungal efficacy and spatial predictions under fluctuating temperatures, indicated that *C. javanica* is suitable for use in different latitudes, although its performance varied at constant temperatures. Wakil et al. (Contribution 11) evaluated the acaricidal efficacy of *B. bassiana* and *M. robertsii* combined with spinosad in both laboratory and greenhouse assays against a tomato pest, the two-spotted spider mite *Tetranychus urticae*. They found a synergistic effect of the combined treatments on tomato plants under greenhouse conditions, which can provide enhanced control of *T. urticae* life stages compared to each treatment applied alone. Liu et al. (Contribution 12) explored the priming effects of *B. bassiana* on tomato plants. They found that the fungus activates phenylpropanoid metabolic pathways, with this modulation being influenced by jasmonate, thereby improving the plant’s resistance to herbivore stress caused by *B. tabaci*. Finally, Almeida et al. (Contribution 13) investigated whether *M. robertsii* can reduce the feeding of the neotropical brown stink bug, *Euschistus heros* using electropenetrography on soybean plants. They found that fungus-treated adults spent significantly less time on probing plants compared to untreated insects and the number of waveform events decreased in the former.

## 3. Conclusions

Entomopathogenic fungi have significant potential as biocontrol agents for managing insect pests through direct infection or the production of indirect metabolites, exhibiting complex interactions with the hosts. This Special Issue covers a diverse range of research, highlighting the richness of the field. Four articles concentrate on enhancing the efficiency of fungi through interactions with lipids and chemical insecticides. Another four articles delve into the functions of genes in *Metarhizium*, aiming to uncover the regulatory mechanisms governing resistance, virulence, or conidiation. Additionally, four articles assess the potential of fungi in insect control, exploring the underlying mechanisms. Finally, one paper specifically examines the infection dynamics of *Beauveria bassiana* in the gut of *Tribolium castaneum*. Overall, each paper introduces novel insights that underscore the significance of this Special Issue, offering readers research focused on multi-level contexts beyond the usual scope, providing a more diverse view of the field.

